# Inhibition of Kynurenine Metabolism and Its Effect in Mitochondrial Function in Hepatocellular Carcinoma

**DOI:** 10.1093/geroni/igaa057.410

**Published:** 2020-12-16

**Authors:** Raul Castro-Portuguez, Samuel Freitas, George Sutphin

**Affiliations:** 1 University of Arizona, Tucson, Arizona, United States; 2 University of Arizona, Tucson, United States

## Abstract

Hepatocellular carcinoma (HCC) is the most prevalent cancer in the liver. The majority of ingested tryptophan is processed in the liver through the kynurenine pathway, the endpoint of which is de novo NAD+ biosynthesis. Dysregulation of tryptophan-kynurenine metabolism and NAD+ synthesis may promote mitochondrial malfunction, tumor reprogramming, and carcinogenesis. Using a publicly available gene expression dataset from liver hepatocellular carcinoma (LIHC) samples available through The Cancer Genome Atlas (TCGA; n = 371), we employed Principal Component Analysis (PCA), hierarchical clustering, gene-pattern expression profiling, and survival analysis to cluster patients and determine overall survival. Our analysis of genes encoding kynurenine pathway enzymes determined that patients with high QPRT expression had a poor prognosis with decreased median survival, with no effect on the maximum survival. There is a significant difference in the survival between patients with high QPRT expression relative to patients with high HAAO/AFMID expression (HR = 1.2, [95% CI 0.5-1.8] P = 0.0181, Gehan-Breslow-Wilcoxon Test). Patients with high QPRT expression have higher survival rates compared with low QPRT expression (HR = 1.4, [95% CI 0.9-2.2] P = 0.0344, Gehan-Breslow-Wilcoxon Test). To test the consequences of kynurenine-pathway inhibition in mitochondrial function and morphology we use 4-Cl-3HAA, an irreversible HAAO inhibitor, and observed a small increase in mitochondrial fragmentation in HepG2 cells after 24 hours of treatment. We conclude that kynurenine metabolism may be useful as a biomarker to predict patient prognosis among HCC patients. In ongoing work, we are testing QPRT inhibitors in cell culture as a potential adjuvant for chemotherapies.

